# Dexmedetomidine improves clinical outcomes in sepsis-induced myocardial injury: a retrospective cohort study

**DOI:** 10.3389/fphar.2024.1529167

**Published:** 2025-01-15

**Authors:** Yuan Liu, Jianjie Ouyang, Cuicui Zhang, Pingping Niu, Baoling Shang, Gengzhen Yao, Yongyong Shi, Xu Zou

**Affiliations:** ^1^ Second Clinical Medical College, Guangzhou University of Chinese Medicine, Guangzhou, Guangdong, China; ^2^ Department of Cardiology, The Second Affiliated Hospital of Guangzhou University of Chinese Medicine, Guangdong Provincial Hospital of Chinese Medicine, Guangzhou, China; ^3^ Department of Anesthesiology, The Second Affiliated Hospital of Guangzhou University of Chinese Medicine, Guangdong Provincial Hospital of Chinese Medicine, Guangzhou, China; ^4^ Guangdong Provincial Hospital of Chinese Medicine, Guizhou Hospital, Guiyang, China

**Keywords:** dexmedetomidine, sepsis, myocardial injury, mortality, MIMIC-IV

## Abstract

**Background:**

The efficacy of dexmedetomidine (DEX) in treating sepsis-induced myocardial injury (SIMI) remains unclear. In this study, we explored the relationship between DEX use and clinical outcomes of patients with SIMI, focusing on the dosage and treatment duration.

**Methods:**

In this retrospective cohort analysis, we identified patients with SIMI from the Medical Information Mart for Intensive Care IV (MIMIC-IV) database and categorized them into the DEX and non-DEX groups based on intensive care unit treatment. The baseline bias was reduced through propensity score matching (PSM). The primary outcome was 28-day mortality, whereas the secondary outcomes were in-hospital mortality and mortality rates at 7 days, 90 days, and 1 year. The association between DEX use and in-hospital mortality was assessed using Kaplan–Meier analysis and Cox proportional hazards models.

**Results:**

After PSM, 373 patients in the DEX group were matched with 579 patients in the non-DEX group to achieve a balanced distribution of the covariates. The Cox regression model demonstrated a significant reduction in the 28-day mortality associated with DEX use, yielding a hazard ratio (HR) of 0.61 (95% confidence interval [CI]: 0.47–0.78, P < 0.001). In-hospital mortality also significantly decreased (HR = 0.43, 95% CI: 0.33–0.57, P < 0.001). Lower mortality rates were observed at 7 days, 90 days, and 1 year. DEX doses >0.4 μg/kg/h, particularly in the range of 0.400–0.612 μg/kg/h, total doses >3.113 mg during hospitalization, and treatment durations exceeding 72 h were associated with improved mortality risk at all intervals. Regarding DEX efficacy at 28 days, our subgroup analyses indicated a significant interaction between the Sequential Organ Failure Assessment score and invasive mechanical ventilation.

**Conclusion:**

DEX administration was associated with improved in-hospital mortality and reduced mortality rates at 7 days, 28 days, 90 days, and 1 year in patients with SIMI. These findings require validation in future studies.

## 1 Introduction

Sepsis, a severe life-threatening condition, has a significant global incidence and mortality rate and poses a grave public health concern (Singer et al., 2016). Despite advancements in healthcare, sepsis still accounts for approximately 20% of global deaths (Rudd et al., 2020). The prevalence of sepsis-induced myocardial injury (SIMI) can be as high as 50% among patients with sepsis, and its occurrence typically signals an unfavorable prognosis (Turner et al., 1999). The mortality rate of patients with SIMI during hospitalization can reach 35%, with a 1-year mortality rate of up to 51% (Frencken et al., 2018). The treatment measures for patients with sepsis have seen some developments; however, those for patients with SIMI are still suboptimal, warranting further improvement. The therapeutic pharmacopeia for SIMI includes vasopressors, inotropes, and recombinant thrombomodulin (Kakihana et al., 2016). However, these treatment modalities are yet to be widely implemented in clinical practice, largely because of the uncertainty regarding their efficacy and potential for significant adverse effects.

Dexmedetomidine (DEX), a selective α2-adrenergic receptor agonist, is commonly used during the critical care of patients in the intensive care unit (ICU) and perioperative period for anesthesia (Bauerschmidt et al., 2023). However, its cardioprotective effects remain unclear. Notably, some studies have suggested that DEX has cardioprotective properties, as evidenced by the reduced myocardial infarction area and improved myocardial contractility in rats after DEX treatment (She et al., 2024). However, other studies have indicated that cardiac magnetic resonance imaging showed a decrease in ventricular systolic function in volunteers after sedation with DEX (Omran et al., 2021). DEX has been used in treating patients with sepsis; however, its impact on their prognosis remains controversial. In certain studies, DEX has been associated with increased survival rates and improved prognosis of patients with sepsis (Chen et al., 2020; Zhang et al., 2022). However, some studies have found that DEX treatment does not significantly impact mortality rates in patients with sepsis (Kawazoe et al., 2017; Ding et al., 2022).

Increasing evidence suggests that DEX possesses anti-inflammatory properties (Xu et al., 2023; Chen et al., 2024; Haotian et al., 2024). Sepsis is characterized by a dysregulated state of organ dysfunction caused by an immune response imbalance to infection (Singer et al., 2016). An exaggerated inflammatory response may play a significant role in SIMI progression (Bi et al., 2022). In addition, studies on the efficacy of DEX in patients with SIMI are limited. Therefore, investigating whether DEX plays a role in improving the outcomes of patients with SIMI is warranted. In this study, we aimed to explore the potential correlation between DEX use and the prognosis of patients with SIMI.

## 2 Materials and methods

### 2.1 Source of the data

In this study, we extracted data from the MIMIC-IV (Medical Information Mart for Intensive Care) version 3.0, accessed at https://physionet.org/content/mimiciv/3.0/ (Johnson et al., 2023). MIMIC-IV consists of a robust, de-identified dataset derived from Beth Israel Deaconess Medical Center emergency department and ICU in Boston, MA. The dataset includes records of over 94,400 patients admitted to the ICU and over 546,000 in-patient admissions documented between 2008 and 2022. The dataset employs anonymous identifiers to safeguard patient privacy. Hence, informed consent was not required. Access authorization for the relevant data in the database was obtained by the author (YL), and the associated course assessment was completed (Certificate No: 65786107).

### 2.2 Population selection criteria and definition

This study’s inclusion criteria were (1) participants aged ≥18 years, (2) meeting Sepsis-3 criteria, (3) fulfilling SIMI criteria (Singer et al., 2016), and (4) ICU stay of over 24 h and up to 100 days.

Per standards and database limits, SIMI was defined as cardiac troponin T (cTnT) levels >0.01 ng/mL, measured within 24 h of ICU admission (Vallabhajosyula et al., 2017; Xie et al., 2021; Dong et al., 2024).

The exclusion criteria were set to remove direct and indirect causes of elevated cTnT levels. Patients with acute coronary syndrome, cardiomyopathy, myocarditis, valvular heart disease, endocarditis, pericarditis, chronic obstructive pulmonary disease, chronic heart failure, prior cardiac surgery or arrest, and severe tachyarrhythmias (including supraventricular tachycardia, ventricular tachycardia, fibrillation, and flutter) were excluded (Pasupula et al., 2023; Dong et al., 2024). We analyzed only the first-admission data for patients with multiple ICU stays. Patients were also excluded if the DEX infusion lasted for <4 h (Morelli et al., 2019; Hu et al., 2022).

### 2.3 Data collection

Data extraction was performed using the Structured Query Language in Navicat Premium software, version 16, utilizing patient stay_id and hadm_id for specificity. The demographic data extracted for analysis include age, sex, and race. Patients’ clinical data extracted for analysis include demographic variables, vital signs, comorbidities, first laboratory test results within 24 h of ICU admission, clinical scoring systems, treatments (including sedative medications), length of stay in the ICU, and hospitalization.

In addition, detailed information regarding DEX administration was collected, including the route of administration, dosage, and duration of infusion.

### 2.4 Outcomes

The primary outcome measure of this study was the 28-day mortality rate, whereas the secondary outcomes included in-hospital mortality. Extended outcomes included mortality rates at 7 days, 90 days, and 1 year.

### 2.5 Statistical analysis

The participants were categorized into two groups according to DEX use: one group received DEX treatment (DEX group), whereas the other group did not (non-DEX group). We used the random forests method for multiple imputations through the Mice package (version 3.16.0) to mitigate potential biases arising from incomplete data (van Buuren and Groothuis-Oudshoorn, 2011). Variables with more than 30% missing data were excluded. Supplementary Table S1 provides comprehensive details regarding the quantity and proportion of missing data. The normality test results showed that not all continuous variables in this study were normally distributed. Consequently, data were presented as median and link ranges, and comparisons within the group were made using the Wilcoxon grid sum test. The variables, classified as frequencies and percentages, were reported, and the differences between the groups were assessed using Pearson’s chi-square test or Fisher’s exact test, if necessary.

Variables demonstrating significant differences at baseline were included as covariates and adjusted using propensity score matching (PSM) to minimize imbalances between the two groups, thereby enhancing the robustness of our findings (Zhang, 2017). A 1:2 nearest-neighbor matching algorithm was utilized, with the caliper width set at 0.1.

We used Kaplan–Meier (KM) analysis to determine the effect of DEX use on the survival of patients with SIMI at various intervals, including in-hospital, 7 days, 28 days, 90 days, and 1 year. Univariate Cox regression assessed how the baseline factors influenced the 28-day mortality, providing hazard ratios (HR) and 95% confidence intervals (CI). Factors with P < 0.05 were selected as covariates for a multivariable Cox regression model. Using the non-DEX group as a reference, we developed three Cox regression models: (1) Model I, which is unadjusted; (2) Model II, adjusted for age, gender, race, systolic blood pressure (SBP), diastolic blood pressure (DBP), and peripheral capillary oxygen saturation (SpO_2_); and (3) Model III, further adjusted for cirrhosis, hypertension, cTnT, white blood cell count, blood urea nitrogen (BUN), serum creatinine, lactate, Sequential Organ Failure Assessment (SOFA) score, Simplified Acute Physiology Score (SAPS) II, invasive mechanical ventilation (IMV), continuous renal replacement therapy, vasopressin, aspirin, beta-blockers, fibrates, and angiotensin converting enzyme inhibitors (ACEI). Model III also explored the dose–response relationship by analyzing the association between DEX dosage, administration duration, and mortality.

Subgroup analyses were conducted to evaluate whether demographic factors, comorbidities, laboratory test results, and other treatments influenced the association between DEX administration and mortality. Statistical significance was defined as a two-sided P-value <0.05. All statistical analyses were performed using the R package (version 4.3.1).

## 3 Results

### 3.1 Baseline characteristics

**FIGURE 1:**
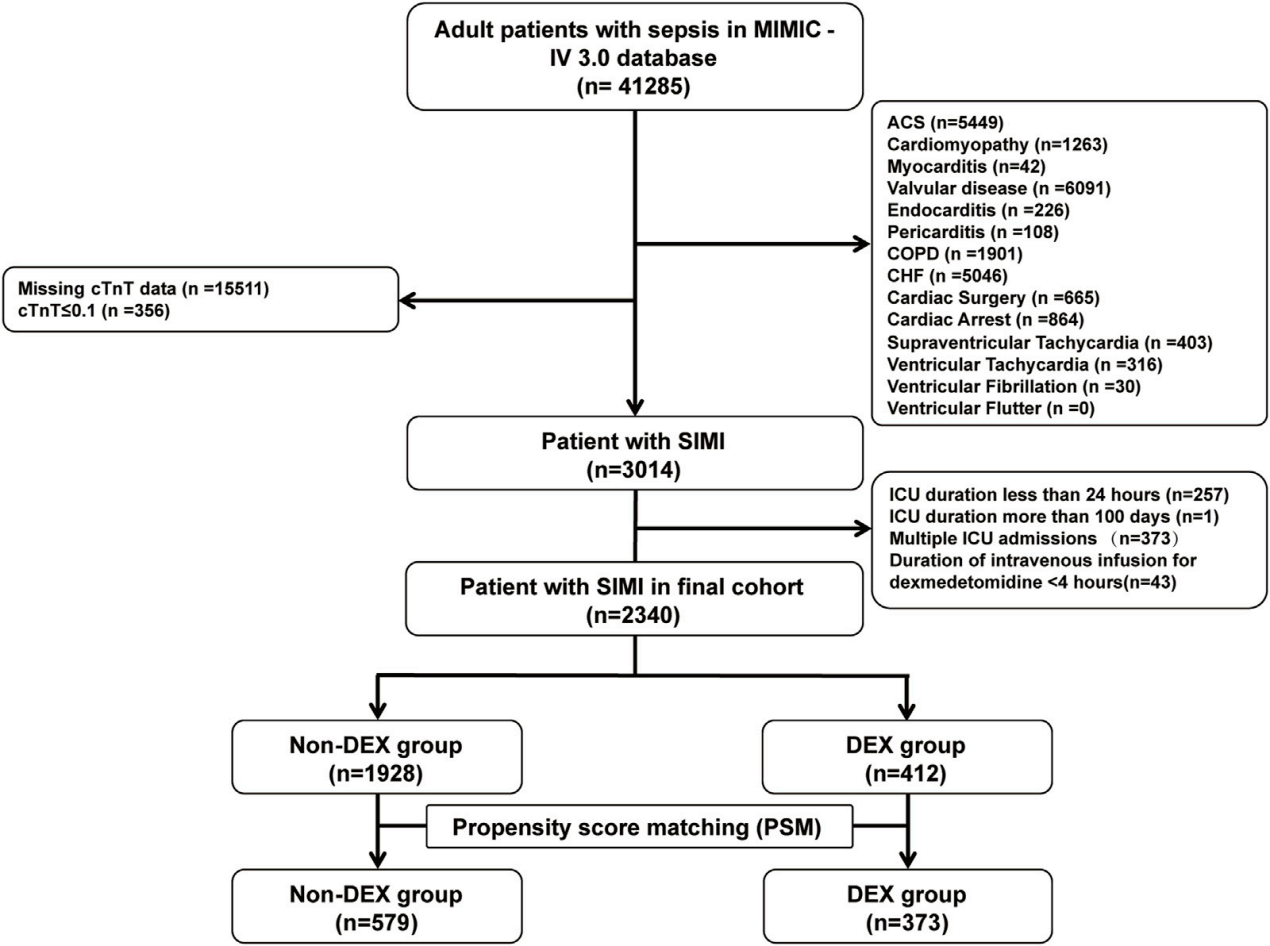
Flow chart of the cohort selection process.

We extracted 41,285 records of patients with sepsis from the MIMIC-IV database, with 3,014 patients meeting the diagnostic criteria for SIMI. After applying the exclusion criteria, 2,340 patients were included in the final analysis, including 412 patients in the DEX group and 1,928 in the non-DEX group (Figure 1). In the original cohort, significant differences were observed between the DEX and non-DEX groups regarding age, race, DBP, body weight, comorbidities (diabetes, hypertension, obesity), laboratory parameters (hemoglobin [Hb], BUN, serum creatinine), SOFA score, and treatments (propofol, fentanyl, midazolam, IMV, continuous renal replacement therapy [CRRT], vasopressin, aldosterone receptor blockers, beta-blockers, fibrates, and statins) (P < 0.05). Table 1 presents a detailed comparison of the baseline characteristics of patients in both groups. Following PSM, 373 patients in the DEX group were matched with 579 patients in the non-DEX group, resulting in a more balanced distribution of covariates between the two groups, with hypertension being the only variable exhibiting a difference. The effectiveness of this matching process was confirmed by assessing the standardized mean differences of the covariates (Supplementary Figure S1).

**TABLE 1:** Baseline characteristics between groups before and after PSM.

Characteristic	Original chort			PSM chort		
	Non-DEX group	DEX group	*P* ^a^	Non-DEX group	DEX group	*P* ^a^
	N = 1,928	N = 412		N = 579	N = 373	
Demographic variables						
Age, Median (Q1, Q3)	69.00 (58.00, 80.00)	63.00 (51.50, 73.00)	<0.001	65.00 (52.00, 76.00)	63.00 (52.00, 73.00)	0.089
Gender, n (%)			0.153			0.891
Male	1,077.00 (55.86%)	246.00 (59.71%)		341.00 (58.89%)	218.00 (58.45%)	
Female	851.00 (44.14%)	166.00 (40.29%)		238.00 (41.11%)	155.00 (41.55%)	
Race, n (%)			<0.001			0.568
White	1,189.00 (61.67%)	221.00 (53.64%)		340.00 (58.72%)	206.00 (55.23%)	
Black	288.00 (14.94%)	46.00 (11.17%)		60.00 (10.36%)	42.00 (11.26%)	
Other	451.00 (23.39%)	145.00 (35.19%)		179.00 (30.92%)	125.00 (33.51%)	
Vital signs on admission, Median (Q1, Q3)						
HR (beats/min)	74.00 (63.00, 85.00)	73.00 (61.50, 84.00)	0.256	75.00 (64.00, 87.00)	73.00 (62.00, 84.00)	0.139
SBP (mmHg)	86.00 (77.00, 96.00)	85.50 (77.00, 94.00)	0.414	86.00 (76.00, 94.00)	85.00 (77.00, 93.00)	0.629
DBP (mmHg)	44.00 (37.00, 51.00)	45.00 (39.00, 52.00)	0.001	45.00 (39.00, 52.00)	45.00 (39.00, 52.00)	0.812
RR (beats/min)	13.00 (10.00, 15.00)	13.00 (10.00, 16.00)	0.948	13.00 (10.00, 15.00)	13.00 (10.00, 16.00)	0.933
SpO2 (%)	92.00 (90.00, 95.00)	92.00 (89.00, 95.00)	0.647	93.00 (90.00, 96.00)	92.00 (89.00, 95.00)	0.101
Weight (kg)	76.00 (63.40, 91.30)	80.00 (68.15, 96.40)	<0.001	78.10 (65.00, 94.00)	80.00 (67.80, 95.40)	0.207
Comorbidity, n (%)						
Diabetes	666.00 (34.54%)	111.00 (26.94%)	0.003	158.00 (27.29%)	95.00 (25.47%)	0.535
Atrial Fibrillation	514.00 (26.66%)	93.00 (22.57%)	0.086	146.00 (25.22%)	84.00 (22.52%)	0.343
Cirrhosis	245.00 (12.71%)	65.00 (15.78%)	0.095	91.00 (15.72%)	63.00 (16.89%)	0.631
Hyperlipidemia	628.00 (32.57%)	136.00 (33.01%)	0.864	179.00 (30.92%)	120.00 (32.17%)	0.684
Hypertension	862.00 (44.71%)	61.00 (14.81%)	<0.001	131.00 (22.63%)	61.00 (16.35%)	0.019
Obesity	169.00 (8.77%)	58.00 (14.08%)	<0.001	58.00 (10.02%)	48.00 (12.87%)	0.172
Biochemistry, Median (Q1, Q3)						
cTnT (ng/mL)	0.06 (0.03, 0.12)	0.06 (0.03, 0.13)	0.117	0.06 (0.03, 0.16)	0.06 (0.03, 0.12)	0.566
CK-MB (IU/L)	4.00 (3.00, 9.00)	5.00 (3.00, 10.00)	0.389	5.00 (3.00, 11.00)	5.00 (3.00, 10.00)	0.181
Hb (g/dL)	9.30 (8.00, 10.90)	9.60 (8.00, 11.50)	0.017	9.40 (8.00, 11.20)	9.40 (7.90, 11.20)	0.722
PLT (K/uL)	166.00 (101.00, 237.00)	152.00 (99.50, 226.50)	0.06	155.00 (89.00, 221.00)	151.00 (97.00, 229.00)	0.934
WBC (K/uL)	9.70 (6.30, 13.90)	9.85 (6.25, 13.50)	0.87	10.10 (6.40, 14.00)	9.70 (6.20, 13.60)	0.381
BUN (mg/dL)	26.00 (16.00, 43.00)	22.00 (14.00, 35.50)	<0.001	22.00 (14.00, 38.00)	22.00 (14.00, 36.00)	0.78
SCR (mg/dL)	1.20 (0.80, 2.30)	1.10 (0.80, 1.90)	0.029	1.10 (0.80, 2.00)	1.10 (0.80, 1.90)	0.696
LAC (mmol/L)	1.50 (1.10, 2.30)	1.50 (1.10, 2.05)	0.594	1.50 (1.10, 2.40)	1.50 (1.10, 2.10)	0.273
Critical assessment on admission, Median (Q1, Q3)						
SOFA	6.00 (4.00, 9.00)	8.00 (5.00, 11.00)	<0.001	8.00 (5.00, 11.00)	8.00 (5.00, 11.00)	0.288
SAPS II	43.00 (34.00, 53.00)	43.00 (35.00, 53.00)	0.608	44.00 (35.00, 56.00)	43.00 (36.00, 54.00)	0.781
Sedative-analgesic medications, n (%)						
Propofol	761.00 (39.47%)	377.00 (91.50%)	<0.001	517.00 (89.29%)	338.00 (90.62%)	0.51
Fentanyl	902.00 (46.78%)	373.00 (90.53%)	<0.001	500.00 (86.36%)	334.00 (89.54%)	0.145
Midazolam	572.00 (29.67%)	217.00 (52.67%)	<0.001	287.00 (49.57%)	193.00 (51.74%)	0.512
Treatment, n (%)						
IMV	677.00 (35.11%)	224.00 (54.37%)	<0.001	332.00 (57.34%)	211.00 (56.57%)	0.814
CRRT	99.00 (5.13%)	36.00 (8.74%)	0.004	56.00 (9.67%)	36.00 (9.65%)	0.992
Vasopressin	297.00 (15.40%)	103.00 (25.00%)	<0.001	142.00 (24.53%)	98.00 (26.27%)	0.544
ACEI	312.00 (16.18%)	77.00 (18.69%)	0.215	83.00 (14.34%)	68.00 (18.23%)	0.108
ARB	29.00 (1.50%)	13.00 (3.16%)	0.022	11.00 (1.90%)	7.00 (1.88%)	0.98
Aspirin	483.00 (25.05%)	114.00 (27.67%)	0.269	146.00 (25.22%)	97.00 (26.01%)	0.785
Beta-blockers	854.00 (44.29%)	217.00 (52.67%)	0.002	293.00 (50.60%)	184.00 (49.33%)	0.701
Fibrates	204.00 (10.58%)	112.00 (27.18%)	<0.001	116.00 (20.03%)	80.00 (21.45%)	0.599
Statins	397.00 (20.59%)	103.00 (25.00%)	0.048	123.00 (21.24%)	84.00 (22.52%)	0.641
Outcomes, n (%)						
In-hospital mortality	498.00 (25.83%)	80.00 (19.42%)	0.006	198.00 (34.20%)	74.00 (19.84%)	<0.001
7-day mortality	245.00 (12.71%)	20.00 (4.85%)	<0.001	83.00 (14.34%)	19.00 (5.09%)	<0.001
28-day mortality	541.00 (28.06%)	88.00 (21.36%)	0.005	191.00 (32.99%)	83.00 (22.25%)	<0.001
90-day mortality	740.00 (38.38%)	127.00 (30.83%)	0.004	246.00 (42.49%)	118.00 (31.64%)	<0.001
1-year mortality	966.00 (50.10%)	157.00 (38.11%)	<0.001	303.00 (52.33%)	146.00 (39.14%)	<0.001
Length of Stay (LOS), Median (Q1, Q3)						
LOS ICU days	3.31 (2.00, 6.77)	10.09 (6.12, 19.46)	<0.001	6.01 (2.83, 11.76)	9.78 (5.95, 16.92)	<0.001
LOS hospital days	9.84 (5.36, 17.96)	20.12 (12.16, 33.06)	<0.001	13.67 (6.89, 23.84)	19.59 (11.46, 32.59)	<0.001

^a^ Wilcoxon rank sum test; Pearson’s Chi-squared test.

Abbreviations: DEX, dexmedetomidine; HR, heart rate; SBP, systolic blood pressure; DBP, diastolic blood pressure; RR, respiratory rate; SpO2, peripheral capillary oxygen saturation; cTnT, Cardiac Troponin T; CK-MB, Creatine Kinase-Muscle/Brain; Hb, hemoglobin; PLT, platelet; WBC, white blood cell; BUN, blood urea nitrogen; SCR, serum creatinine; LAC, lactic acid; SOFA, sequential organ failure assessment; SAPSII, Simplified Acute Physiology Score II; IMV, invasive mechanical ventilation; CRRT, continuous renal replacement therapy; ACEI, angiotensin-converting enzyme inhibitor; ARB, Angiotensin II, receptor blocker; LOS, length of stay.

Table 1 presents the baseline statistical analysis of patients’ survival data. Compared to the non-DEX group, the DEX group exhibited lower in-hospital, 7-day, 28-day, 90-day, and 1-year mortality rates, as well as longer total hospital stays and ICU length of stay. These differences were significant (P < 0.05).

### 3.2 Kaplan–Meier analysis

**FIGURE 2:**
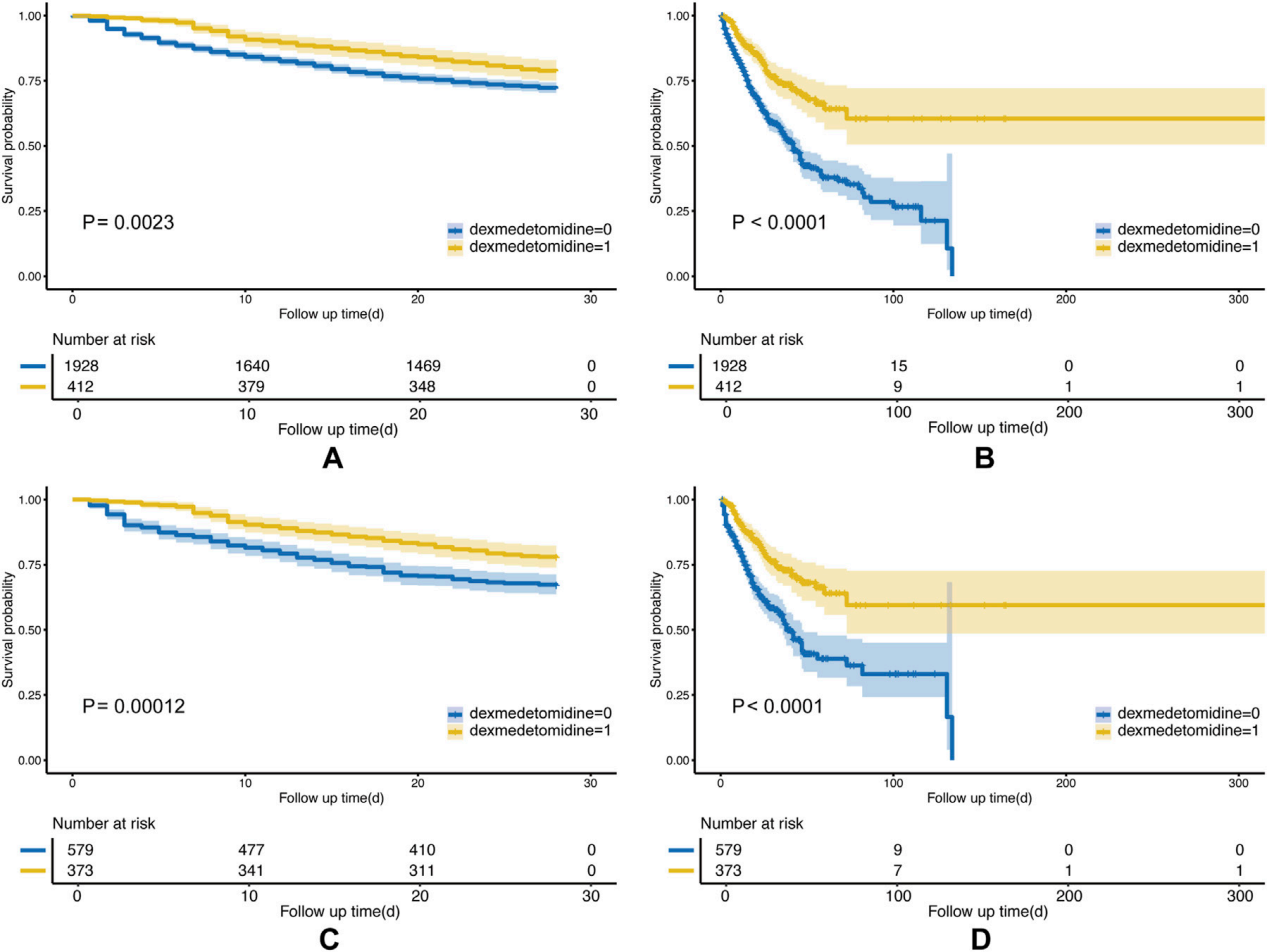
Kaplan–Meier (KM) curves of the Non-DEX group and DEX group. The **(A)** 28-day mortality before PSM; **(B)** in-hospital mortality before PSM; **(C)** 28-day mortality after PSM; and **(D)** in-hospital mortality after PSM.

Compared with the non-DEX group, in the DEX group, the KM survival curves revealed a significantly lower 28-day mortality rate (P = 0.0023; Figure 2A). This finding persisted even after PSM (P = 0.00012; Figure 2B). In-hospital mortality was also significantly reduced in the DEX group in pre- and post-PSM cohorts (P < 0.0001; Figures 2C, D). In addition, KM analyses of the 7-day, 90-day, and 1-year mortality rates in both cohorts indicated lower mortality rates in the DEX group (Supplementary Figure S2).

### 3.3 Association between DEX use and mortality

For the post-PSM cohort, univariate Cox regression showed that DEX use was linked to a 39% reduction in the 28-day mortality risk (HR: 0.61, 95% CI: 0.47–0.78, P < 0.001). Factors such as age, sex, race, SBP, DBP, SpO_2_, cirrhosis, hypertension, cTnT, white blood cell count, BUN, lactate, SOFA score, SAPS II, IMV, CRRT, vasopressin, ACEI, aspirin, beta-blockers, and fibrates also significantly affected the mortality risk (Supplementary Table S2). These variables were incorporated as covariates in the multivariable Cox regression analysis.

Multivariate Cox regression analysis highlighted the effect of DEX on mortality (Table 2). In the crude analysis, DEX was significantly associated with reduced in-hospital mortality (HR = 0.43, 95% CI: 0.33–0.57, P < 0.001). The 7-day mortality rate decreased by 67% (HR = 0.33, 95% CI: 0.20–0.55, P < 0.001), with a 39% reduction in the 28-day mortality (HR = 0.61, 95% CI: 0.47–0.78, P < 0.001). The 90-day mortality rate decreased by 34% (HR = 0.66, 95% CI: 0.53–0.82, P < 0.001) and 1-year mortality rate by 35% (HR = 0.65, 95% CI: 0.53–0.79, P < 0.001). After adjusting for multiple confounding factors, all models consistently demonstrated a significant association between DEX administration and reduced mortality (P < 0.001), suggesting that DEX administration is associated with a lower mortality risk in hospitalized patients.

**TABLE 2:** Association between DEX use and mortality.

Mortality	In-hospital mortality		7-day mortality		28-day mortality		90-day mortality		1-year mortality	
	HR (95%CI)	*P*	HR (95%CI)	*P*	HR (95%CI)	*P*	HR (95%CI)	*P*	HR (95%CI)	*P*
Model I	0.43 (0.33, 0.57)	<0.001	0.33 (0.20, 0.55)	<0.001	0.61 (0.47, 0.78)	<0.001	0.66 (0.53, 0.82)	<0.001	0.65 (0.53, 0.79)	<0.001
Model II	0.43 (0.33, 0.56)	<0.001	0.31 (0.19, 0.52)	<0.001	0.59 (0.46, 0.77)	<0.001	0.65 (0.52, 0.81)	<0.001	0.65 (0.54, 0.80)	<0.001
Model III	0.41 (0.31 0.55)	<0.001	0.34 (0.21, 0.57)	<0.001	0.57 (0.44, 0.74)	<0.001	0.63 (0.50, 0.79)	<0.001	0.43 (0.33, 0.57)	<0.001

HR, hazard ratio; CI, confidence interval.

Model I: Crude.

Model II: Adjust: Age, Gender, Race, SBP, DBP, SpO2.

Model III: Adjust: Age, Gender, Race, SBP, DBP, SpO2, Cirrhosis, Hypertension, cTnT, WBC, BUN, LAC, SOFA, SAPSII, IMV, CRRT, Vasopressin, ACEI, Aspirin, Beta-blockers, Fibrates.

Abbreviations: SBP, systolic blood pressure; DBP, diastolic blood pressure; SpO2, peripheral capillary oxygen saturation; cTnT, Cardiac Troponin T; WBC, white blood cells; BUN, blood urea nitrogen; LAC, lactic acid; SOFA, sequential organ failure assessment; SAPSII, Simplified Acute Physiology Score II; IMV, invasive mechanical ventilation; CRRT, continuous renal replacement therapy; ACEI, angiotensin-converting enzyme inhibitors.

### 3.4 Duration and doses of DEX and their relationship with all-cause mortality

Prolonged administration of DEX for over 72 h was associated with a significant reduction in the risks of in-hospital, 7-day, 28-day, 90-day, and 1-year mortality rates. In addition, DEX administration for 24–72 h demonstrated its effectiveness in decreasing the risks of in-hospital, 7-day, and 1-year mortality. By contrast, short-term use of DEX (4–24 h) was significantly correlated with reduced risks of in-hospital and 7-day mortality; however, it showed no significant impact on mortality rates at 28 days, 90 days, and 1 year (Table 3).

**TABLE 3:** Association between duration and doses of DEX and mortality.

Mortality	In-hospital mortality		7-day mortality		28-day mortality		90-day mortality		1-year mortality	
	HR (95%CI)	*P*	HR (95%CI)	*P*	HR (95%CI)	*P*	HR (95%CI)	*P*	HR (95%CI)	*P*
Dexmedetomidine duration (h)										
Non-DEX	References		References		References		References		References	
4–24 h	0.46 (0.27, 0.79)	0.005	0.21 (0.07, 0.69)	0.01	0.64 (0.40, 1.02)	0.061	0.80 (0.55, 1.17)	0.3	0.80 (0.58, 1.12)	0.2
24–72 h	0.49 (0.28, 0.84)	0.01	0.4 (0.16, 0.99)	0.047	0.8 (0.51, 1.25)	0.3	0.72 (0.49, 1.08)	0.12	0.63 (0.43, 0.92)	0.017
>72 h	0.33 (0.21, 0.52)	<0.001	0.11 (0.03, 0.46)	0.002	0.35 (0.21, 0.58)	<0.001	0.43 (0.28, 0.65)	<0.001	0.39 (0.27, 0.58)	<0.001
Dexmedetomidine dose (ug/kg/h)										
Non-DEX	References		References		References		References		References	
<0.400	0.45 (0.27, 0.75)	0.002	0.23 (0.07, 0.74)	0.014	0.7 (0.45, 1.10)	0.12	0.81 (0.56, 1.19)	0.3	0.83 (0.59, 1.16)	0.3
0.400–0.612	0.36 (0.22, 0.59)	<0.001	0.21 (0.08, 0.60)	0.003	0.47 (0.29, 0.75)	0.002	0.57 (0.39, 0.84)	0.004	0.5 (0.35, 0.71)	<0.001
>0.612	0.41 (0.25, 0.67)	<0.001	0.23 (0.07, 0.74)	0.014	0.51 (0.31, 0.83)	0.008	0.52 (0.34, 0.80)	0.003	0.49 (0.33, 0.73)	<0.001
Total dose of dexmedetomidine used during hospitalization (mg)										
Non-DEX	References		References		References		References		References	
<0.414	0.34 (0.17, 0.68)	0.002	0.2 (0.05, 0.83)	0.027	0.52 (0.29, 0.94)	0.03	0.64 (0.41, 1.02)	0.061	0.66 (0.44, 0.99)	0.044
0.414–3.113	0.51 (0.31, 0.82)	0.005	0.37 (0.16, 0.85)	0.02	0.8 (0.53, 1.19)	0.3	0.84 (0.59, 1.18)	0.3	0.76 (0.55, 1.04)	0.088
>3.113	0.36 (0.23, 0.55)	<0.001	0.11 (0.03, 0.45)	0.002	0.39 (0.24, 0.62)	<0.001	0.45 (0.30, 0.67)	<0.001	0.41 (0.28, 0.60)	<0.001

HR, hazard ratio; CI, confidence interval.

Adjust: Age, Gender, Race, SBP, DBP, SpO2, Cirrhosis, Hypertension, cTnT, WBC, BUN, LAC, SOFA, SAPSII, IMV, CRRT, Vasopressin, ACEI, Aspirin, Beta-blockers, Fibrates.

Abbreviations: SBP, systolic blood pressure; DBP, diastolic blood pressure; SpO2, peripheral capillary oxygen saturation; cTnT, Cardiac Troponin T; WBC, white blood cells; BUN, blood urea nitrogen; LAC, lactic acid; SOFA, sequential organ failure assessment; SAPSII, Simplified Acute Physiology Score II; IMV, invasive mechanical ventilation; CRRT, continuous renal replacement therapy; ACEI, angiotensin-converting enzyme inhibitors.

The interquartile range of DEX dosage (33%–66%) was 0.400–0.612 μg kg^−1^ h^−1^. Compared with the non-DEX group, DEX dosages ranging from 0.400 to 0.612 μg kg^−1^ h^−1^ and above 0.612 μg kg^−1^ h^−1^ significantly reduced the risks of in-hospital, 7-day, 28-day, 90-day, and 1-year mortality. Specifically, for in-hospital and 28-day mortality, the effect of the 0.400–0.612 μg kg^−1^ h^−1^ dosage on reducing mortality risk was the most pronounced, with 64% and 53% reductions, respectively. Moreover, DEX dosages exceeding 0.612 μg kg^−1^ h^−1^ demonstrated significant reductions in mortality risk across all time points. By contrast, DEX dosages <0.400 μg kg^−1^ h^−1^ significantly impacted in-hospital and 7-day mortality, with no significant effects observed on 28-day, 90-day, or 1-year mortality (Table 3).

The interquartile range (33%–66%) of the total DEX dose used during hospitalization was 0.414–3.113 mg. DEX was associated with significant reductions in in-hospital, 7-day, 28-day, 90-day, and 1-year mortality when the total DEX dose was ≥3.113 mg. For in-hospital and 28-day mortality, total doses >3.113 mg reduced mortality by 64% and 61%, respectively, and total doses <0.414 mg reduced mortality by 66% and 48%, respectively. by contrast, the total dose between 0.414 and 3.113 mg was significantly different only for in-hospital and 7-day mortality, but no significant difference was observed for 28-day, 90-day, and 1-year mortality (Table 3).

### 3.5 Subgroup analysis

**FIGURE 3:**
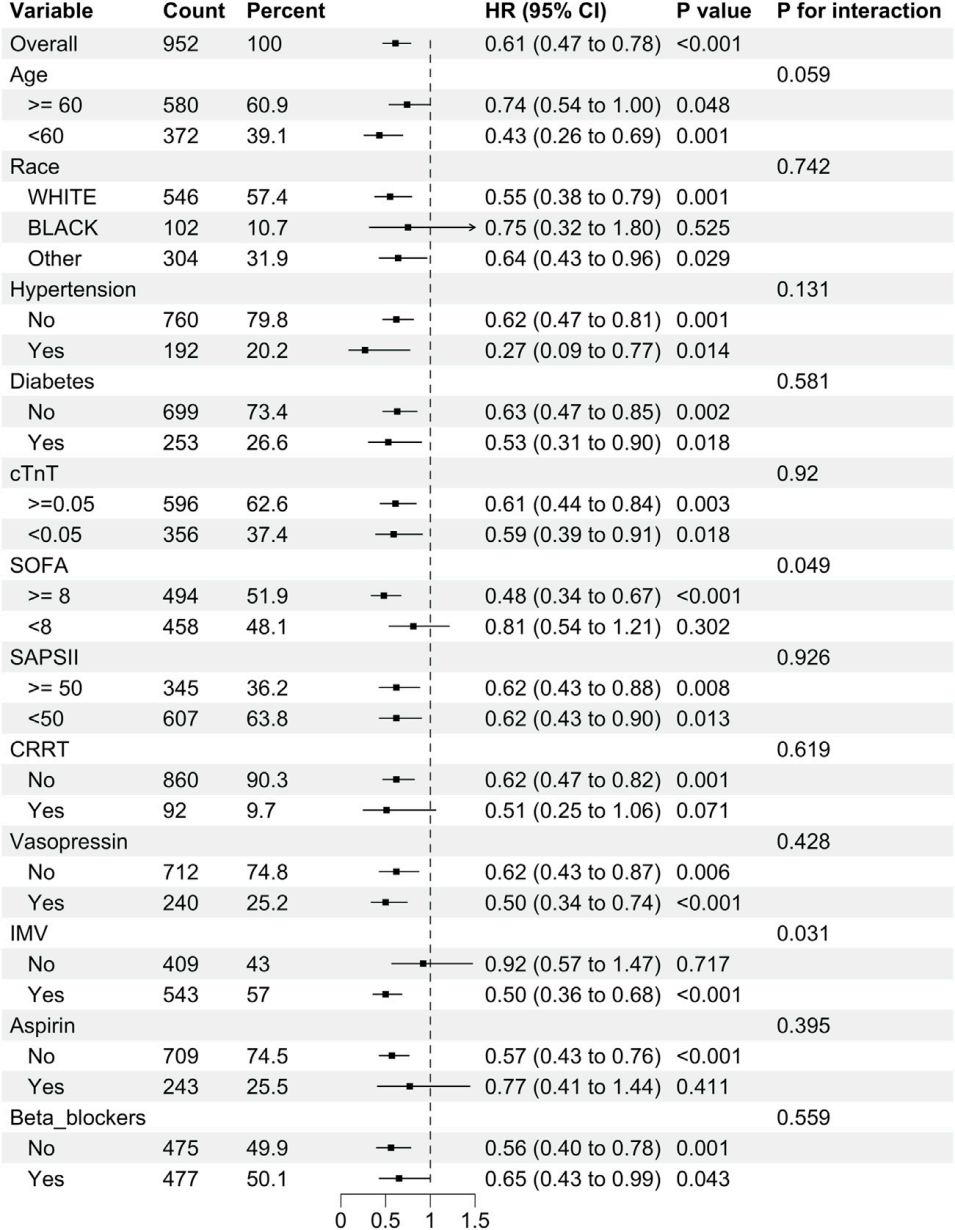
Subgroup analysis of the relationship between DEX use and 28-day mortality in SIMI patients.

We performed subgroup analyses to investigate the relationship between DEX administration and 28-day mortality (Figure 3). The results indicated that DEX exhibited a significant protective effect for patients with a SOFA score of ≥8 (P < 0.001) and those requiring IMV (P < 0.001). Conversely, no significant effect was observed for patients with a SOFA score of <8 or those not receiving IMV (P > 0.05, interaction P < 0.05). Furthermore, in patients with SIMI who underwent CRRT and those treated with aspirin, there was a tendency for DEX to provide protection against 28-day mortality; however, this was not significant (P > 0.05, interaction P > 0.05).

Furthermore, we examined the association between DEX administration and in-hospital mortality across the different subgroups (Supplementary Figure S3). The results demonstrated that DEX administration conferred varying degrees of potential benefits across the subgroups, with consistent results (interaction P > 0.05). The subgroup analyses, thus, revealed heterogeneity in the effects of DEX on 28-day mortality among various patient populations while consistently demonstrating a protective effect on in-hospital mortality. These findings have important implications for personalized treatment strategies for patients with SIMI.

## 4 Discussion

Our findings demonstrated that DEX administration was significantly associated with a reduction in the risk of in-hospital, 7-day, 28-day, 90-day, and 1-year mortality rates among patients with SIMI. However, patients receiving DEX had longer hospital stays and ICU lengths of stay than those not receiving DEX. Furthermore, upon analyzing the administration protocol of DEX, our findings indicated that duration of administration exceeding 72 h, medium doses of DEX >0.4 μg kg^−1^ h^−1^ (especially 0.400–0.612 μg kg^−1^ h^−1^), and total DEX dose >3.113 mg were significantly associated with improved mortality risk at various time points.

Before this study, no research had examined the correlation between DEX use and clinical mortality outcomes of patients with SIMI. To our knowledge, this is the first study to show that DEX therapy can improve mortality rates in patients with SIMI at 7 days and 28 days. High-sensitivity troponin I, creatine kinase-MB, and myoglobin (MYO) are significantly reduced in patients with SIMI on days 3 and 7 after DEX therapy (Si et al., 2024). B-type natriuretic peptide levels and left ventricular ejection fraction (LVEF) also significantly improved on days 3 and 7 after DEX therapy. Procalcitonin, interleukin-1β, and tumor necrotic factor-α (TNF-α) were significantly reduced in patients with SIMI on day 7 after DEX treatment. This suggests that the significant decrease in the levels of myocardial injury markers, heart failure indices, and inflammatory markers in patients with SIMI after DEX treatment may be a potential reason for the improvement in the short-term mortality caused by DEX in patients with SIMI. DEX treatment reduces in-hospital and 30-day mortality in patients with acute myocardial infarction, and the efficacy was possibly mediated by a reduction in leukocyte levels (Liu et al., 2024). DEX treatment may improve short-term survival and ameliorate endothelial damage in patients with sepsis-induced coagulopathy. DEX administration may also improve the prognosis of patients with sepsis-induced coagulopathy by reducing platelet activation, by suppressing inflammatory markers (Huang et al., 2024). These studies suggest that DEX reduces short-term mortality in patients with myocardial damage and may involve decreasing the level of inflammation, which reduces myocardial damage, attenuates endothelial injury, and improves cardiac function. In addition, DEX improves the prognosis of patients with myocardial damage. Notably, we discovered that DEX treatment not only improved the short-term survival of patients with SIMI but also improved their 90-day and 1-year mortality and long-term prognosis.

However, the precise mechanism by which DEX exerts its therapeutic effects on sepsis-induced myocardial damage remains unclear. A possible explanation is that DEX modulates the immune response, impacting the progression of sepsis-related myocardial damage through anti-inflammatory actions and alleviation of mitochondrial injury pathways (Lankadeva et al., 2021; Raupach et al., 2021). DEX exerts anti-inflammatory effects in clinical use and animal experiments (Slim et al., 2024). SIMI is associated with inflammation, and anti-inflammatory agents effectively treat SIMI (Wang et al., 2021). Thus, DEX may ameliorate myocardial injury by counteracting inflammation and oxidative stress (He et al., 2023). Mitochondrial dysfunction is a key factor in the pathophysiology of SIMI (Bi et al., 2022). Mitochondria regulate myocardial metabolism and inflammation, and mitochondrial dysfunction critically affects myocardial function (Martin et al., 2019). Mitochondrial dysfunction can cause cardiomyocyte death through apoptosis or necrosis (Kuroshima et al., 2024). In an animal model of myocardial ischemia/reperfusion, DEX alleviated myocardial mitochondrial apoptosis through a pathway involving the lncRNA HCP5/miR-29a/MCL1 axis and activation of Janus kinase 2/signal transducer and activator of transcription 3 signaling (Deng et al., 2022). DEX improves SIMI by alleviating sepsis-induced myocardial mitochondrial dysfunction. No animal model research has validated this theory, and further studies are required to confirm it.

Our findings indicate a significant correlation between DEX treatment and a decreased mortality risk in patients with SIMI during hospitalization and at 7 days, 28 days, 90 days, and 1 year. DEX provides cardioprotection for patients undergoing cardiac surgery with extracorporeal circulation by reducing postoperative troponin levels (Chen et al., 2022). Intraoperative DEX infusion can improve the prognosis of cardiac patients undergoing surgery and enhance their 5-year survival rate (Peng et al., 2021). DEX administration can enhance long-term survival rates among older patients admitted to the ICU after non-cardiac surgery (Zhang et al., 2019). Furthermore, it can improve patients’ cognitive levels, quality of life, psychological well-being, and social interactions. DEX may also prolong hospital and ICU stays while reducing the 7-day, 90-day, and 1-year mortality rates of patients with SIMI. The prolonged hospitalization and ICU stay may be ascribed to its sedative properties, which require an extended period of monitoring and evaluation during the recuperative phase (Yavarovich et al., 2019). DEX has demonstrated protective effects against various cardiac diseases and improved outcomes of patients with heart conditions (Chen et al., 2022). DEX use exhibited a potentially advantageous effect on the survival rates of patients with SIMI across different time intervals, although the reasons and mechanisms require further investigation.

DEX duration and dosage analysis revealed significant associations with mortality outcomes at various time points. We found that extending the duration of DEX administration (beyond 72 h) significantly reduced in-hospital, 7-day, 28-day, 90-day, and 1-year mortality rates. Studies have suggested a significant correlation between the dose or timing of DEX administration and reduced mortality rates in young and older patients with sepsis requiring IMV (Zhao et al., 2024). Experimental animal studies have demonstrated that DEX improves mortality rates in rats with sepsis in a dose-dependent manner, reduces inflammatory cytokine levels, ameliorates lactic acidosis, and shows a positive correlation between survival rate and DEX dosage (Ma et al., 2018). Moreover, the dose–response analysis in this study indicated that moderate (0.400–0.612 μg kg^−1^ h^−1^) and high (exceeding 0.612 μg kg^−1^ h^−1^) DEX doses were linked to decreased mortality risk at all assessed time points, with the moderate dose showing the most pronounced effect on mortality reduction. In addition, we analyzed the relationship between the total DEX dose and the prognosis of patients with SIMI. In this study, we showed that the total DEX dose used, when <0.414 mg or >3.113 mg, could significantly increase the 28-day survival rate of patients with SIMI, and the protective effect was more significant when the total dose was >3.113 mg. A higher total DEX dose showed a significant protective effect on the short- and long-term survival of patients with SIMI. However, evidence suggests that the administration of high doses of DEX may increase the incidence of adverse reactions, including hypotension and bradycardia (Fang et al., 2023). Additional research is needed to elucidate the most appropriate dose and duration of DEX administration for patients with SIMI.

Consistent with the results of previous studies, our subgroup analyses revealed that DEX had a notable protective effect in patients with SIMI requiring IMV. Patients with SIMI who require IMV typically exhibit severe respiratory failure and significantly elevated levels of inflammatory markers (Zhao et al., 2024). DEX administration reduces C-reactive protein (CRP) and procalcitonin levels in patients with sepsis requiring mechanical ventilation, improves albumin levels, and alleviates inflammation (Ohta et al., 2020). Furthermore, DEX therapy may be beneficial for hypoxic pulmonary vasoconstriction and ventilation-perfusion mismatch (Jain et al., 2021). Thus, DEX treatment may have significant prognostic implications in patients with SIMI requiring mechanical ventilation. In addition, we observed that DEX showed potential efficacy in patients with a SOFA score of ≥8. We hypothesize that this is due to the severe organ dysfunction indicated by a SOFA score >8, which is associated with more pronounced heart failure and extensive myocardial injury than those in patients with a SOFA score of <8, likely accompanied by more severe inflammatory responses (Beesley et al., 2018). In addition, among patients with SIMI who required CRRT and those treated with aspirin, DEX showed a trend of protection against 28-day mortality; however, this was not significant. Evidence suggests that DEX treatment may shorten the duration of CRRT and reduce the incidence of acute kidney injury in patients receiving intensive care (Liu et al., 2020). Aspirin may also reduce the risk of death in patients with SIMI (Dong et al., 2024). We believe caution should be exercised when interpreting the efficacy of DEX in patients with SIMI treated with CRRT or aspirin, as the number of patients in this subgroup differed significantly from that in the control group. Therefore, future clinical trials with larger sample sizes are needed to clarify the effect of DEX on improving the outcomes of patients with SIMI in different subgroups.

Our study had certain limitations. First, owing to the study’s retrospective observational design, we recognize that although we utilized PSM and multivariable analyses comprehensively to reduce the influence of confounding variables, it was not feasible to adjust for all variables. Consequently, some potential confounders may have been overlooked. Second, although we attempted to exclude most patients with underlying cardiovascular diseases, those included in the study cohort may not have had SIMI as their primary diagnosis. Third, in addition to clinical mortality outcomes, cardiac function indicators and inflammatory markers (cTnT, BNP, N-terminal pro-B-type natriuretic peptide, MYO, LVEF, CRP, high-sensitivity CRP, interleukin-6, TNF-α) are important outcome variables for patients with SIMI. However, owing to the significant amount of missing data for these indicators (as detailed in Supplementary Table S1), we could not analyze them as outcome measures, and this may have influenced our results. Fourth, our study could only establish associations and not imply causation. Fifth, this single-center retrospective cohort study did not include cohorts from Asian populations. Future multicenter, large-sample, prospective studies are needed to validate our findings.

## 5 Conclusion

DEX treatment can improve in-hospital mortality rates in patients with SIMI and reduce mortality rates at 7 days, 28 days, 90 days, and 1 year. These findings provide a basis for clinical decision-making regarding DEX administration. However, further validation using randomized controlled trials is required.

## Data Availability

The datasets presented in this study can be found in online repositories. The names of the repository/repositories and accession number(s) can be found below: https://physionet.org/content/mimiciv/3.0/.
