# Supplementary material for: Dexmedetomidine improves clinical outcomes in sepsis-induced myocardial injury: a retrospective cohort study
Source: Front Pharmacol. 2025 Jan 15;15:1529167. doi: 10.3389/fphar.2024.1529167 (PMC11774859; doi:10.3389/fphar.2024.1529167)
Supplement: Supplementary file 1 [file Supplementaryfile1.docx]

**Supplementary Material:**


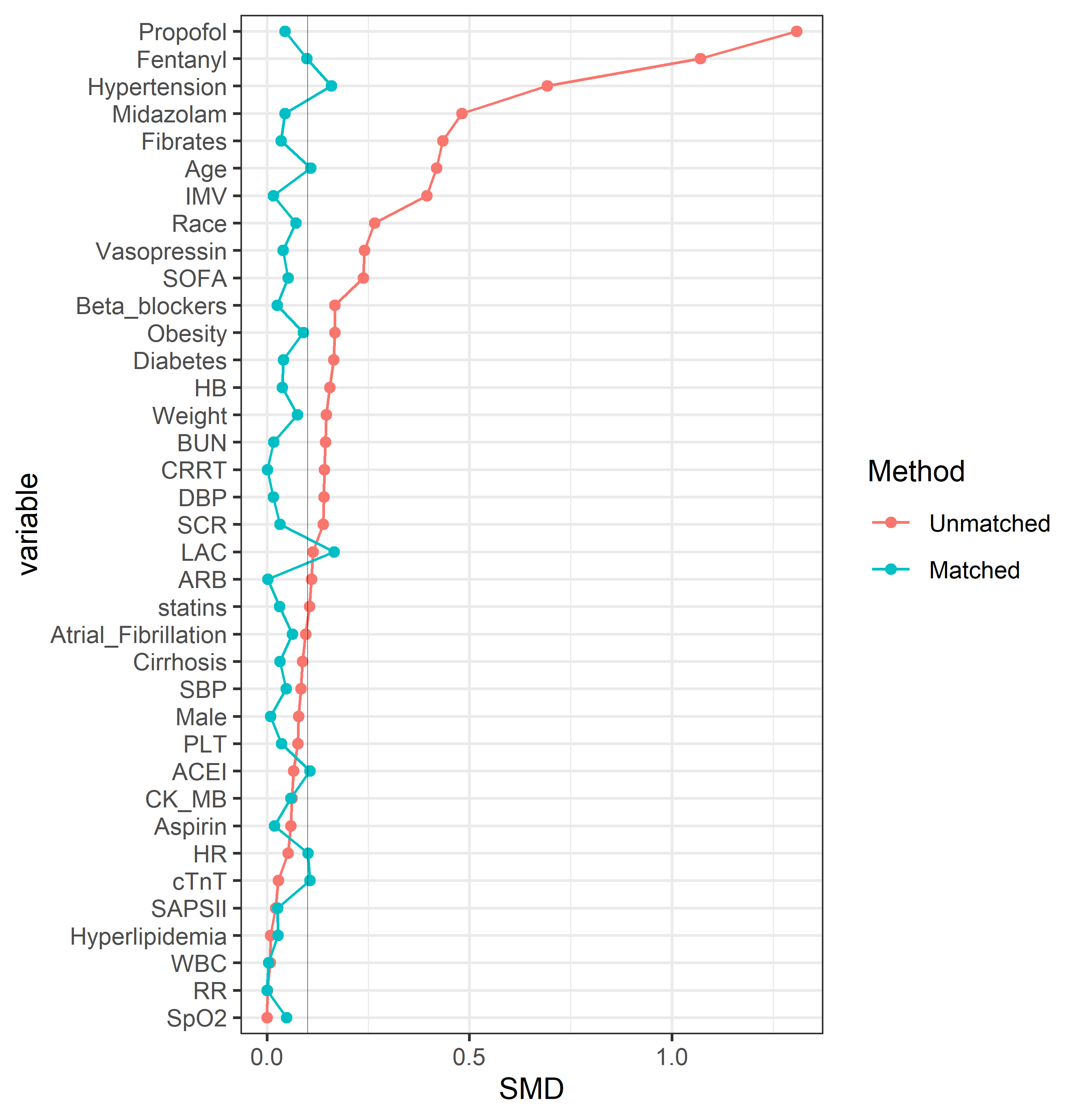


**Supplementary Figure S1.** Comparison of SMD before and after PSM.

**Abbreviations:** IMV, Invasive Mechanical Ventilation; SOFA, Sequential Organ Failure Assessment; HB, Hemoglobin; BUN, Blood Urea Nitrogen; CRRT, Continuous Renal Replacement Therapy; DBP, Diastolic Blood Pressure; SCR, Serum Creatinine; LAC, Lactic Acid; ARB, Angiotensin II Receptor Blocker; SBP, Systolic Blood Pressure; PLT, Platelet; ACEI, Angiotensin-Converting Enzyme Inhibitor; CK_MB, Creatine Kinase-Muscle/Brain; HR, Heart Rate; cTnT, Cardiac Troponin T; SAPSII, Simplified Acute Physiology Score II; WBC, White Blood Cell; RR, Respiratory Rate; SpO2, Peripheral Capillary Oxygen Saturation.


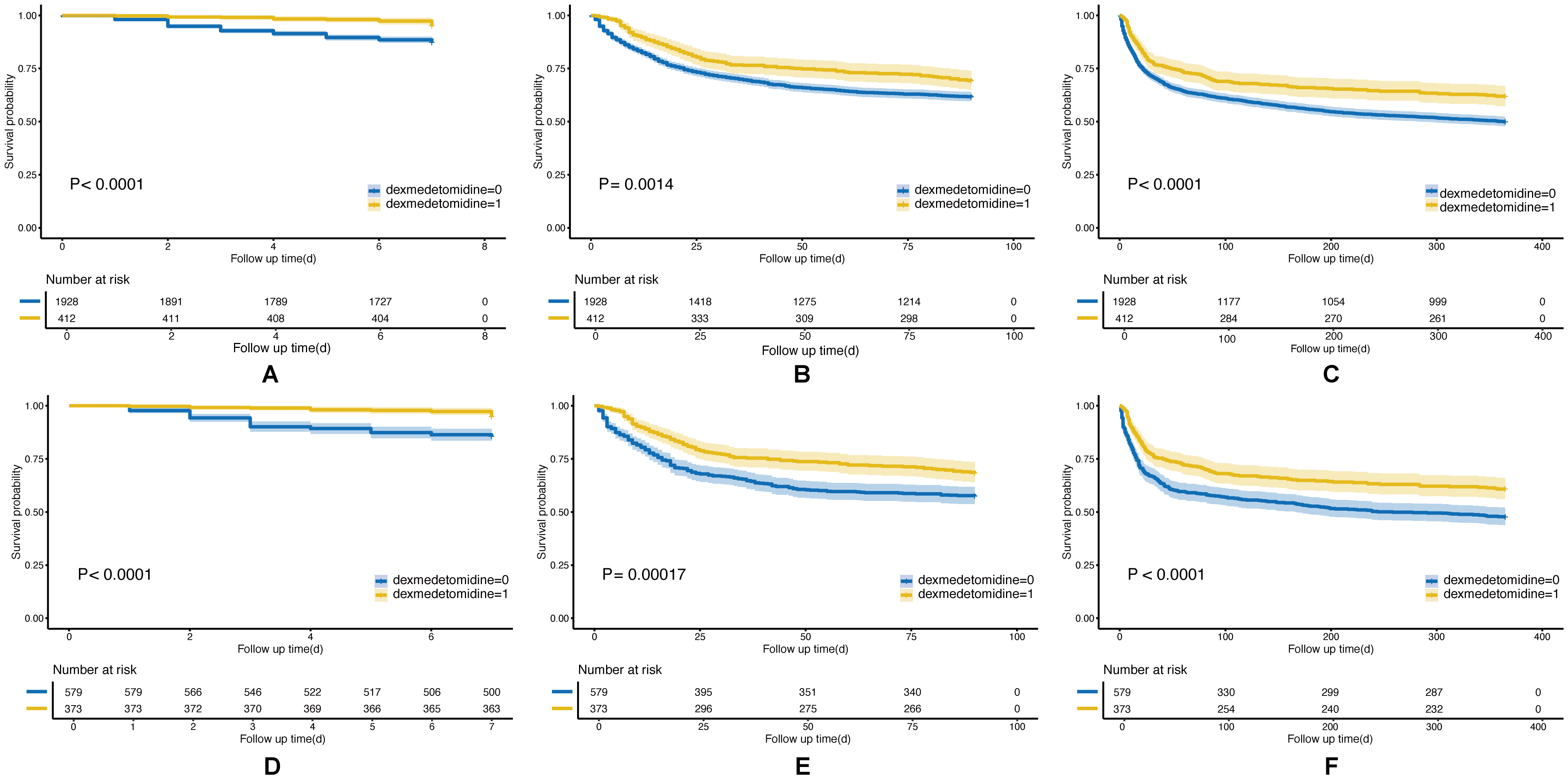


**Supplementary Figure S2.** K Kaplan–Meier (KM) curves of the Non-DEX group and DEX group. (A) 7-day mortality before PSM; (B) 90-day mortality before PSM; (C) 1-year mortality before PSM; (D) 7-day mortality after PSM; € 90-day mortality after PSM; (F) 1-year mortality after PSM.

**Abbreviations:** DEX, Dexmedetomidine; PSM, Propensity Score Matching.


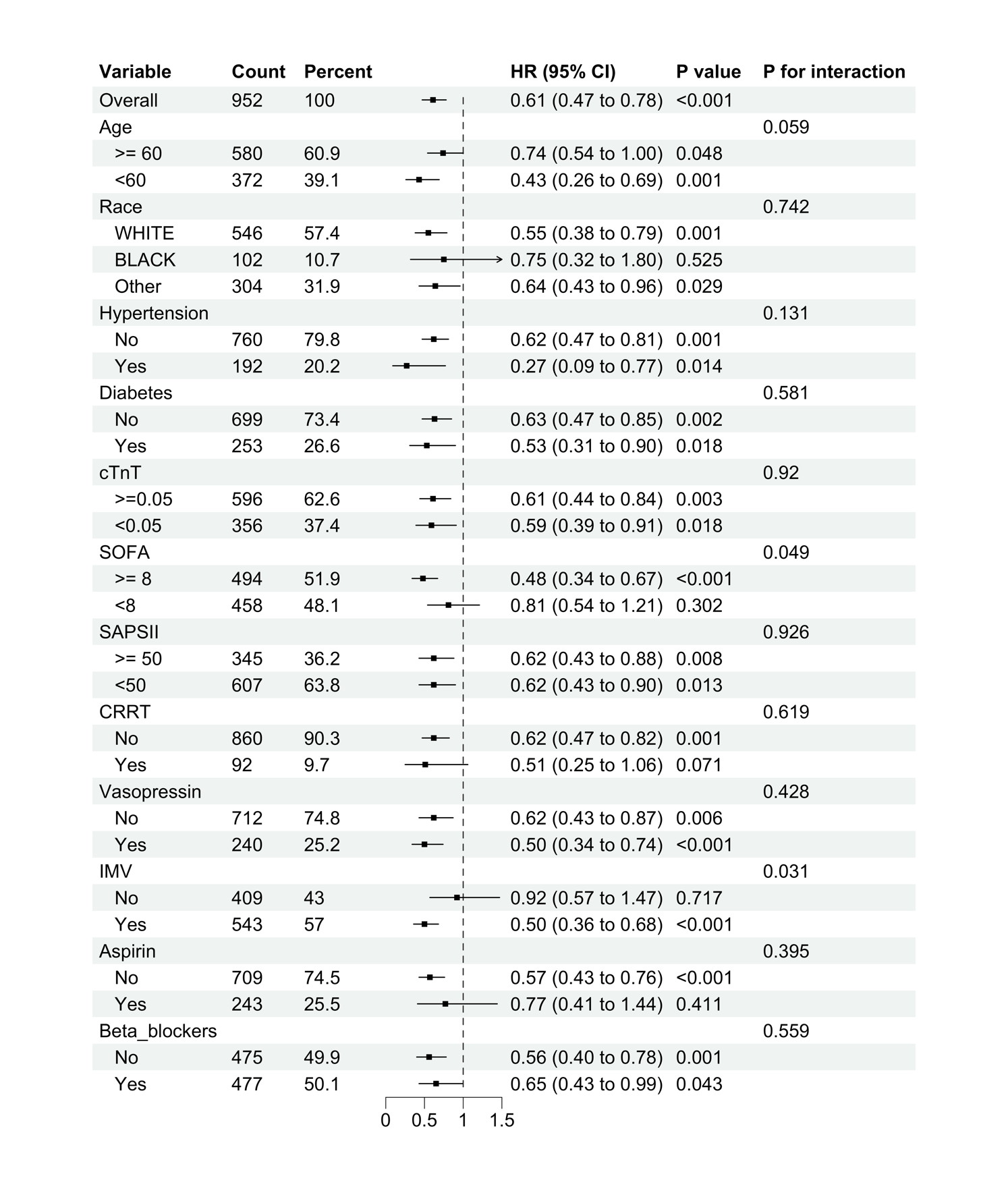


**Supplementary Figure S3.** Subgroup analysis of the relationship between DEX use and in-hospital mortality in SIMI patients.

**Abbreviations:** cTnT, Cardiac Troponin T; SOFA, Sequential Organ Failure Assessment; SAPSII, Simplified Acute Physiology Score II; CRRT, Continuous Renal Replacement Therapy; IMV, Invasive Mechanical Ventilation.

| **Variable** | **MissingCount** | **MissingPercent (%)** |
| --- | --- | --- |
| MYO | 2340 | 100 |
| BNP | 2340 | 100 |
| IL-6 | 2340 | 100 |
| TNF-a | 2340 | 100 |
| hsCRP | 2339 | 99.96 |
| NTproBNP | 2059 | 87.99 |
| CRP | 2030 | 86.75 |
| LvEF | 1864 | 79.66 |
| Height | 989 | 42.26 |
| CK | 864 | 36.92 |
| CK-MB | 642 | 27.44 |
| LAC | 588 | 25.13 |
| Weight | 62 | 2.65 |
| RR | 7 | 0.3 |
| HB | 6 | 0.26 |
| HR | 5 | 0.21 |
| SBP | 5 | 0.21 |
| DBP | 5 | 0.21 |
| SpO2 | 5 | 0.21 |
| PLT | 5 | 0.21 |
| WBC | 5 | 0.21 |
| BUN | 5 | 0.21 |
| SCR | 5 | 0.21 |
| Age | 0 | 0 |
| Male | 0 | 0 |
| Race | 0 | 0 |
| Diabetes | 0 | 0 |
| Atrial Fibrillation | 0 | 0 |
| Cirrhosis | 0 | 0 |
| Hyperlipidemia | 0 | 0 |
| Hypertension | 0 | 0 |
| Obesity | 0 | 0 |
| cTnT | 0 | 0 |
| SOFA | 0 | 0 |
| SAPSII | 0 | 0 |
| IMV | 0 | 0 |
| CRRT | 0 | 0 |
| Vasopressin | 0 | 0 |
| Propofol | 0 | 0 |
| Fentanyl | 0 | 0 |
| Midazolam | 0 | 0 |
| ACEI | 0 | 0 |
| ARB | 0 | 0 |
| Aspirin | 0 | 0 |
| Beta blockers | 0 | 0 |
| Fibrates | 0 | 0 |
| statins | 0 | 0 |
| dexmedetomidine | 0 | 0 |
| LOS ICU days | 0 | 0 |
| LOS hospital days | 0 | 0 |

**Supplementary Table S1.** Missing number for included variables in the datasets.

**Abbreviations:** MYO, Myoglobin; BNP, B-type Natriuretic Peptide; IL-6, Interleukin-6; TNF-a, Tumor Necrosis Factor-alpha; hsCRP, High-Sensitivity C-Reactive Protein; NTproBNP, N-terminal pro-B-type Natriuretic Peptide; CRP, C-Reactive Protein; LvEF, Left Ventricular Ejection Fraction;

CK, Creatine Kinase; CK-MB, Creatine Kinase-Muscle/Brain; LAC, Lactic Acid; RR, Respiratory Rate; HB, Hemoglobin; HR, Heart Rate; SBP, Systolic Blood Pressure; DBP, Diastolic Blood Pressure; SpO2, Peripheral Capillary Oxygen Saturation; PLT, Platelets; WBC, White Blood Cells; BUN, Blood Urea Nitrogen; SCR, Serum Creatinine; cTnT, Cardiac Troponin T; SOFA, Sequential Organ Failure Assessment; SAPSII, Simplified Acute Physiology Score II; IMV, Invasive Mechanical Ventilation; CRRT, Continuous Renal Replacement Therapy; ACEI, Angiotensin-Converting Enzyme Inhibitors; ARB, Angiotensin II Receptor Blockers; LOS, Length of Stay.

.

| **Characteristic** | **HR (95%CI)** | **P** |
| --- | --- | --- |
| dexmedetomidine | 0.61 (0.47-0.78) | **<0.001** |
| Age | 1.00 (1.00-1.00) | **<0.001** |
| Gender | 0.76 (0.60-0.96) | **0.024** |
| Race | 1.20 (1.00-1.30) | **0.012** |
| HR | 1.00 (1.00-1.00) | 0.32 |
| SBP | 0.99 (0.98-1.00) | **0.009** |
| DBP | 0.98 (0.97-1.00) | **0.007** |
| RR | 1.00 (1.00-1.10) | 0.079 |
| SpO2 | 0.97 (0.95-0.98) | **<0.001** |
| Weight | 1.00 (0.99-1.00) | 0.22 |
| Diabetes | 0.96 (0.73-1.30) | 0.77 |
| Atrial Fibrillation | 1.20 (0.93-1.60) | 0.15 |
| Cirrhosis | 1.50 (1.20-2.10) | **0.003** |
| Hyperlipidemia | 0.90 (0.70-1.20) | 0.44 |
| Hypertension | 0.49 (0.34-0.71) | **<0.001** |
| Obesity | 0.94 (0.64-1.40) | 0.75 |
| cTnT | 1.20 (1.10-1.30) | **0.002** |
| CK-MB | 1.00 (1.00-1.00) | 0.62 |
| HB | 0.95 (0.90-1.00) | 0.069 |
| PLT | 1.00 (1.00-1.00) | 0.23 |
| WBC | 1.00 (1.00-1.00) | **<0.001** |
| BUN | 1.00 (1.00-1.00) | **<0.001** |
| SCR | 1.00 (0.93-1.10) | 0.95 |
| LAC | 1.30 (1.20-1.40) | **<0.001** |
| SOFA | 1.10 (1.00-1.10) | **<0.001** |
| SAPSII | 1.00 (1.00-1.00) | **<0.001** |
| IMV | 2.40 (1.80-3.10) | **<0.001** |
| CRRT | 1.50 (1.10-2.20) | **0.018** |
| Vasopressin | 2.90 (2.30-3.70) | **<0.001** |
| Propofol | 0.93 (0.63-1.40) | 0.7 |
| Fentanyl | 1.20 (0.80-1.70) | 0.43 |
| Midazolam | 0.96 (0.76-1.20) | 0.72 |
| ACEI | 0.34 (0.21-0.54) | **<0.001** |
| ARB | 0.52 (0.17-1.60) | 0.26 |
| Aspirin | 0.49 (0.35-0.68) | **<0.001** |
| Beta-blockers | 0.56 (0.44-0.71) | **<0.001** |
| Fibrates | 0.68 (0.49-0.94) | **0.019** |
| statins | 0.73 (0.54-1.00) | 0.051 |

**Supplementary Table S2.** Univariate Cox proportional hazards models of 28-day mortality.

**Abbreviations:** HR, Heart Rate; SBP, Systolic Blood Pressure; DBP, Diastolic Blood Pressure; RR, Respiratory Rate; SpO2, Peripheral Capillary Oxygen Saturation; cTnT, Cardiac Troponin T; CK-MB, Creatine Kinase-Muscle/Brain; HB, Hemoglobin; PLT, Platelets; WBC, White Blood Cells; BUN, Blood Urea Nitrogen; SCR, Serum Creatinine; LAC, Lactic Acid; SOFA, Sequential Organ Failure Assessment; SAPSII, Simplified Acute Physiology Score II; IMV, Invasive Mechanical Ventilation; CRRT, Continuous Renal Replacement Therapy; ACEI, Angiotensin-Converting Enzyme Inhibitors; ARB, Angiotensin II Receptor Blockers.
